# The Formation Criteria of LPSO Phase in Type I LPSO Mg-Y-X Alloy from the Perspective of Liquid–Solid Correlation

**DOI:** 10.3390/ma17205032

**Published:** 2024-10-15

**Authors:** Tangpeng Ma, Jin Wang, Jixue Zhou, Jingyu Qin, Kaiming Cheng, Huan Yu, Dongqing Zhao, Huabing Yang, Chengwei Zhan, Guochen Zhao, Xinxin Li

**Affiliations:** 1 Shandong Provincial Key Laboratory of High Strength Lightweight Metallic Materials, Advanced Materials Institute, Qilu University of Technology (Shandong Academy of Sciences), Jinan 250014, China; 2 School of Materials Science and Engineering, Dalian University of Technology, Dalian 116024, China; 3 Key Laboratory for Liquid-Solid Structural Evolution and Processing of Materials, Ministry of Education, Shandong University, Jinan 250061, China; 4 School of Materials Science and Engineering, Liaocheng University, Liaocheng 252000, China

**Keywords:** LPSO, formation criterion, liquid Mg-Y-X alloy, *ab-initio* molecular dynamics

## Abstract

The formation criteria of the LPSO phase are important for the design of long-period stacking-ordered (LPSO) Mg alloys. This work focuses on Type I LPSO Mg-Y-X alloys and attempts to explore the formation criteria of the LPSO phase from the perspective of liquid-solid correlation. With the aid of *ab-initio* molecular dynamics simulation, liquid Mg-Y-X alloys are investigated to obtain the common liquid characteristics from the reported Type I LPSO Mg-Y-X alloys. Following the liquid characteristics, a new Type I LPSO alloy, i.e., Mg-Y-Au, is experimentally confirmed. The discovery of a new Type I LPSO alloy supports liquid–solid correlation, and hence, the formation criteria of the LPSO phase in Type I LPSO alloys can be developed based on the common liquid characteristics of Type I LPSO Mg-Y-X alloys as follows: X should result in the reduction in equilibrium volume and cohesive energy; Y should repulse Y and be attracted by both Mg and X, and X should be repulsed by both Mg and X; X should enhance the threefold and fourfold symmetries and weaken the fivefold and sixfold ones so that the local structural symmetries are distributed close to liquid pure Mg.

## 1. Introduction

The long-period stacking-ordered (LPSO) phase has been shown to significantly improve the strength and ductility of Mg alloys and sparked a continuing wave of research in the last two decades [1,2,3,4]. Stimulated by the excellent mechanical properties, a series of LPSO Mg-RE-X alloys is discovered. For the reported LPSO Mg-RE-X alloys, RE covers Y, Gd, Dy, Ho, and Er, and X covers Al, Co, Ni, Cu, and Zn, such as Mg-Y-Al [5], Mg-Y-Co [6], Mg-Y-Ni [7], Mg-Y-Cu [8], Mg-Y-Zn [9], Mg-Gd-Al [10], Mg-Dy-Ni [11], Mg-Ho-Cu [12], and Mg-Er-Cu [13]. Upon extensive research, an issue becomes more and more important for understanding LPSO Mg alloys and further guiding the alloy design: What kind of element combination supports the formation of the LPSO phase in a Mg alloy?

After experimentally investigating the structure of Mg_97_Zn_1_RE_2_ (RE = Y, La, Ce, Pr, Nd, Sm, Eu, Gd, Tb, Dy, Ho, Er, and Yb) alloys, Kawamura et al. [14] first proposed the characteristics of the constituents of LPSO Mg-RE-Zn alloys: negative mixing enthalpy of each pair, large difference in atomic size, hexagonal closed packed (hcp) structure at room temperature, and large solid solubility limit above several at. % in Mg. Then, in the following work where the LPSO phase is observed in a Mg-Y-Cu alloy, Kawamura et al. [15] modified the description of the characteristics to make it suitable for LPSO Mg-RE-X alloys as extensive as possible. The modified description is as follows: Mg, RE, and X have a negative mixing enthalpy for each pair, in particular, the mixing enthalpy of the RE-X pair is large and negative; RE elements have hcp structure at room temperature and large solid solubility limits above approximately 3.75 at. % in Mg; and the atomic size of X and the RE element is considerably different from that of Mg. Kawamura et al.’s description resembles the Hume–Rothery rules [16] and can be expected to be a set of formation criteria of the LPSO phase for exploring new LPSO Mg alloys.

In terms of the formation criteria, the characteristics of the constituents of the LPSO Mg-RE-X alloys proposed by Kawamura et al. are still not precise enough, although the description has been further optimized by specifying that the atomic size of RE is 8.4% to 11.9% larger than Mg [17]. For example, the mixing enthalpy of the RE-X pair and the atomic size of X are not clearly defined. On the other hand, density functional theory (DFT) calculation can achieve the precise definition of a parameter, thereby naturally rising to be an anticipated method in exploring the formation criteria of the LPSO phase.

Thermodynamic stability is a crucial factor for phase formation. In an effort to describe the thermodynamic stability, Saal and Wolverton [18] defined a stability parameter ΔH_stab_, which is the energy of the LPSO structure with respect to the energy of the convex hull at the LPSO phase’s composition. With the aid of DFT calculation, Saal and Wolverton [19] examined the stability parameter of “interstitial” LPSO structural model [20] in 140 Mg-X^L^-X^S^ alloys, where X^L^ denotes an alloying element larger than Mg and X^S^ denotes an alloying element smaller than Mg. This work successfully predicted the LPSO Mg-Y-Al alloy before it was experimentally confirmed [21] and suggested that the non-REs, such as Ca, Sr, and Th, are promising X^L^ elements in terms of forming a stable LPSO phase, particularly with X^S^ = Zn. However, Liu and Li [22] subsequently argued that Ca and Sr do not have the capability to form an LPSO phase with Al, Zn, Cu, and Ni in a Mg alloy based on their proposed trimer model because the areas and cohesive energies of Mg-Ca-X^S^ and Mg-Sr-X^S^ trimer are respectively much larger and higher than the standard Mg-Y-Zn trimer.

As the DFT study on LPSO structures progresses, more formation criteria of the LPSO phase are proposed [23,24,25]. However, only the stability parameter ΔH_stab_ achieved the discovery of a new LPSO Mg alloy. Saal and Wolverton had mentioned [19] that the reliability of thermodynamic stability evaluated by ΔH_stab_ is determined by the availability of crystal structures in the Inorganic Crystal Structure Database (ICSD), and it is always a lower bound because completing ICSD is a long-term work. Therefore, exploring multiple formation criteria of the LPSO phase independent from the ICSD based on more extensive DFT works is currently necessary to promote the discovery of new LPSO Mg alloys.

In our previous work [26], the chemical environment and structural ordering in a liquid Mg-Y-Zn system were investigated by *ab-initio* molecular dynamics (AIMD) simulation, and two important liquid characteristics were revealed: the strong interaction between Y and Zn causes the superior chemical short-range order (CSRO) that Y atoms are attracted by Zn, and the distribution of local structural symmetry in a liquid ternary Mg-Y-Zn alloy is much closer to pure Mg compared with binary Mg-Y and Mg-Zn alloys. Liquid is the parent state for the casting process; what is more, the Mg-Y-Zn alloy belongs to Type I LPSO alloys, where the LPSO phases can be formed during the casting process [9]. From the perspective of liquid–solid correlation, the previous work further inspires us to consider whether there are liquid characteristics shared by Type I LPSO Mg alloys and whether the common liquid characteristics can serve as the formation criteria of the LPSO phase in Type I LPSO Mg alloys.

**Figure 1:**
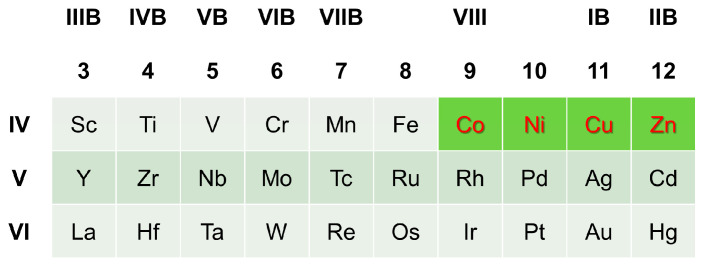
The considered X elements in Mg-Y-X alloys. The X elements of the reported Type I LPSO alloys are highlighted in red.

In this work, AIMD investigations were performed on liquid Mg-Y-X alloys (X elements contain the transition-metal elements in Figure 1). The liquid characteristics of Mg-Y-X alloys were examined in three aspects: equilibrium volume and cohesive energy, chemical short-range order, and local structural symmetry. Then, the common liquid characteristics of Type I LPSO Mg-Y-X alloys were obtained from the reported Type I LPSO Mg-Y-X alloys (X = Co, Ni, Cu, Zn) [6,7,8,9]. Following the obtained common liquid characteristics, the candidate Type I LPSO Mg-Y-X alloys were suggested, and an as-cast Mg-Y-Au alloy selected from the candidates was prepared. By the scanning electron microscope (SEM) and transmission electron microscope (TEM), the Type I LPSO Mg-Y-Au alloy was firstly confirmed. The discovery of a new LPSO alloy supports that the common liquid characteristics are capable of serving as the formation criteria of the LPSO phase, and liquid–solid correlation can be expected to develop into an important principle for alloy design.

## 2. Methodology

### 2.1. Calculation Details

The AIMD simulations were performed using the Vienna *ab-initio* simulation software package (VASP, version 5.4) [27,28] based on DFT [29]. The interactions between ionic cores and valence electrons were treated by the projector augmented wave (PAW) potential [30], and the exchange-correlation energies were determined via the Perdew–Burke–Ernzerhof (PBE) functional [31] within the generalized gradient approximation (GGA). Only Γ-point was used for sampling the Brillouin zone. The NVT ensemble condition was imposed by the Nosé–Hoover thermostat [32,33] in the dynamics process, and the velocity Verlet algorithm was employed to integrate the Newton equation of motion with a time step of 3 fs.

The initial configuration of each liquid alloy was a cubic supercell in which 400 atoms are randomly distributed using the Monte Carlo algorithm, and the numbers of Mg, Y, and X atoms are 320, 40, and 40, respectively. The simulated temperature was set as 1023 K, approximately 100 K higher than the melting point of pure Mg. To fully equilibrate the liquid structures under the average external pressure close to 0 (±1 kbar), all the configurations were firstly relaxed for 3 ps. Then, additional simulations of 15 ps were followed to provide the samples for data analysis.

The CSRO and local structural symmetry were examined based on the largest standard cluster (LSC) algorithm [34]. By counting the near neighbors around the central atom in the LSC, the coordination number can be obtained, and the CSRO was evaluated by the Warren–Cowley parameter. The Warren–Cowley parameter is defined by the following equation [26]:

(1) $$\alpha_{A-B}=1-\frac{Z_{A-B}}{c_{B}Z_{A}}$$

where *c_B_* is the concentration of *B*, and *Z_A_* and *Z_A_*_−*B*_ are the total coordination number and partial coordination number, respectively. *α_A_*_−*B*_ = 0 represents the random distribution of *B* around *A*. *A* positive value of *α_A_*_−*B*_ indicates the repulsion of *A* to *B*, otherwise it means attraction.

The local structural symmetry was determined from the center-neighbor-subclusters in the LSC [34], which are described by four indices as *ijkl* [34,35]. *i* is 1 if the atoms comprising a root pair are near neighbors, *j* is the number of common-near-neighbors shared by the root pair, *k* is the number of bonds between common-near-neighbors, and *l* is the number of bonds in the longest continuous chain formed by the *k* bonds. According to the definition, the local structural symmetry can be represented by the index *j*.

### 2.2. Experimental Procedures

The as-cast Mg-Y-Au alloys were prepared with the nominal composition of Mg_92.5_Y_5_Au_2.5_ (at. %). The raw materials included pure Mg (99.99 wt.%), pure Au (99.99 wt.%), and Mg-30Y (wt.%) intermediate alloy. The cast ingot was produced by vacuum induction melting in an Al_2_O_3_ crucible, followed by cooling to room temperature under the protective atmosphere of Ar (99 vol%).

The microstructure was observed by scanning electron microscopy (SEM, Zeiss EVO MA10, Oberkochen, Germany), and the local chemical composition was semi-quantitatively analyzed by an energy dispersive spectrometer (EDS, Oxford INCA X-Max 50, Oxfordshire, UK) attached to the SEM. To determine the structure of the LPSO phase, the selected area electron diffraction (SAED) pattern and high-resolution TEM image were obtained by transmission electron microscopy (TEM, FEI Tecnai G^2^ F20, Hillsboro, OR, USA) operated at an accelerating voltage of 200 kV.

## 3. Results and Discussion

### 3.1. The Common Liquid Characteristics of Type I LPSO Mg-Y-X Alloys

**Figure 2:**
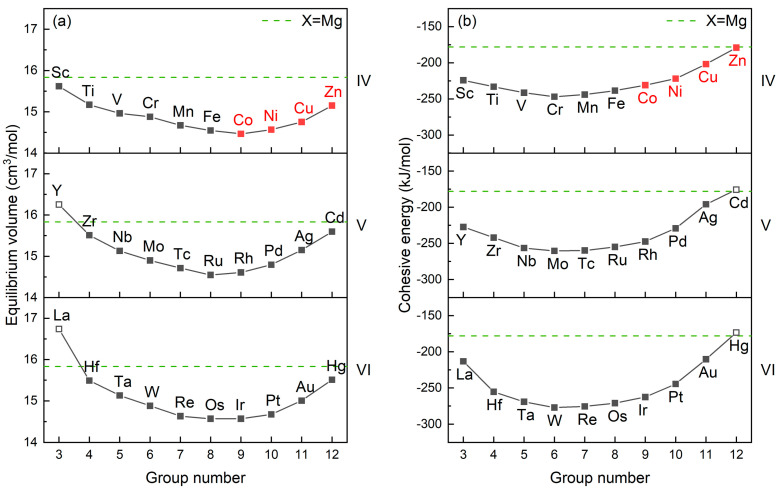
The equilibrium volumes (**a**) and cohesive energies (**b**) of liquid Mg_320_Y_40_X_40_ alloys, which are compared with those of Mg_360_Y_40_.

The equilibrium volumes and cohesive energies of liquid Mg_320_Y_40_X_40_ alloys are calculated and compared with those of Mg_360_Y_40_ alloys as shown in Figure 2. Along the group number, the evolutions of equilibrium volume and cohesive energy follow the patterns consistent with those for Mg-based solid solutions [36,37], suggesting that such evolution patterns should be universal for Mg-based systems whether in solid or liquid state. Upon the patterns, the reported Type I LPSO Mg-Y-X alloys (X = Co, Ni, Cu, Zn) as well as the majority of the considered Mg_320_Y_40_X_40_ alloys have a smaller equilibrium volume and lower cohesive energy compared with Mg_360_Y_40_. We also calculated the equilibrium volume and cohesive energy of liquid pure Mg at 1023 K (15.68 cm^3^/mol and −125.49 kJ/mol), in which the equilibrium volume is smaller than that of Mg_360_Y_40_ and the cohesive energy is higher. The comparisons illustrate that the addition of Y in liquid Mg leads to the enlargement in equilibrium volume and reduction in cohesive energy, and X provides the opposite effect of Y on the equilibrium volume and further reduces the cohesive energy for Type I LPSO Mg-Y-X alloys.

**Figure 3:**
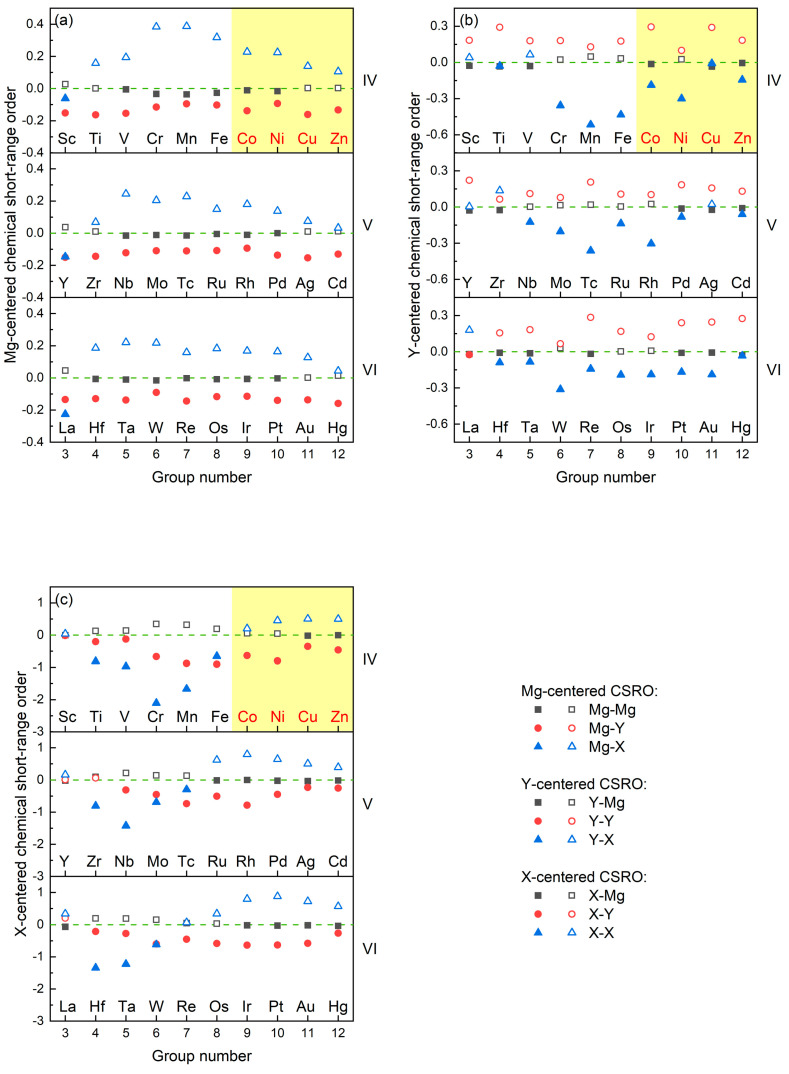
The Warren–Cowley parameters of liquid Mg_320_Y_40_X_40_ alloys: (**a**) Mg-centered CSRO, (**b**) Y-centered CSRO, (**c**) X-centered CSRO.

The CSRO in liquid Mg_320_Y_40_X_40_ alloys is evaluated by Warren–Cowley parameters as exhibited in Figure 3. Among the Warren–Cowley parameters, *α_Mg_*_-*Y*_ < 0, *α_Mg_*_-*X*_ > 0, *α_Y_*_-*Y*_ > 0, *α_Y_*_-*X*_ < 0, *α_X_*_-*Y*_ < 0, and *α_X_*_-*X*_ > 0 are shared by the reported Type I LPSO Mg-Y-X alloys (X = Co, Ni, Cu, Zn). The Warren–Cowley parameters for liquid Mg_320_Y_40_Zn_40_ alloys were also calculated in our previous work [26]. However, *α_Y_*_-*X*_ > 0 was given, which is contrary to that in this work. The inconsistency reflects that *α_Y_*_-*X*_ is insufficiently strong to be stable in the liquid dynamics process. Therefore, the common CSRO in Type I LPSO Mg-Y-X alloys should be determined by *α_Mg_*_-*Y*_ < 0, *α_Mg_*_-*X*_ > 0, *α_Y_*_-*Y*_ > 0, *α_X_*_-*Y*_ < 0, and *α_X_*_-*X*_ > 0, which correspond to the attraction of Mg to Y, the repulson of Mg to X, the repulson of Y to Y, the attraction of X to Y, and the repulsion of X to X, respectively.

**Figure 4:**
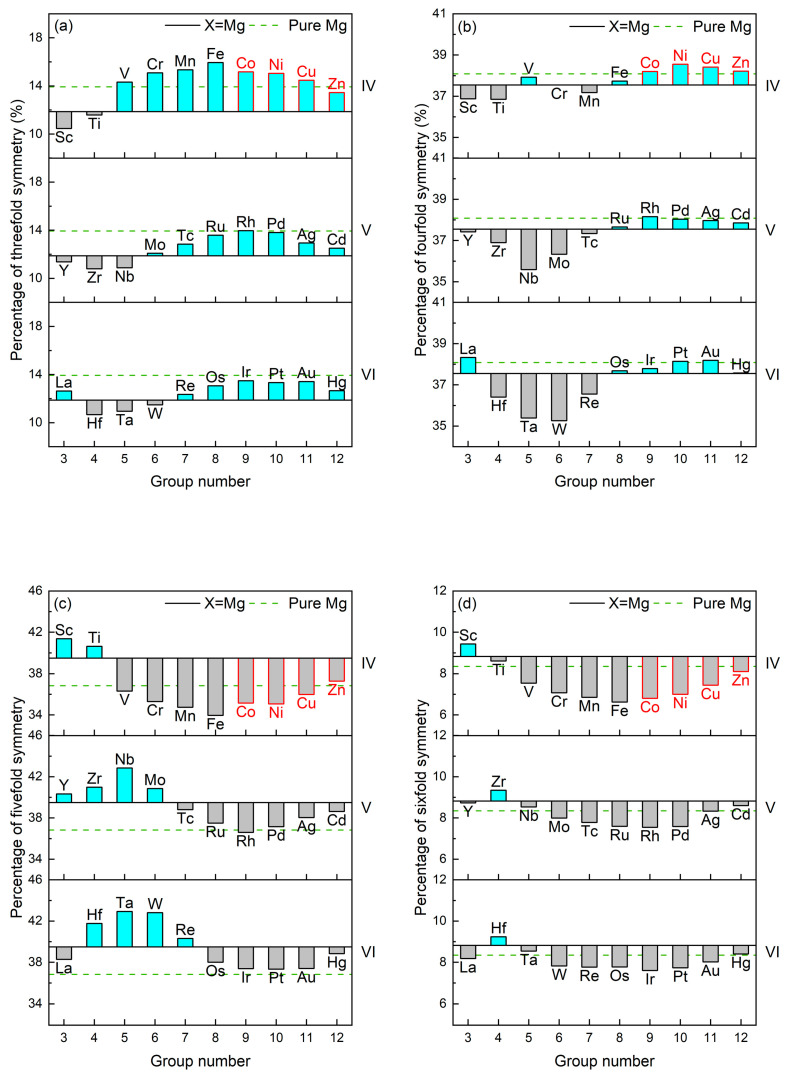
The changes in percentages of main local structural symmetries in liquid Mg_320_Y_40_X_40_ alloys relative to the counterparts in liquid Mg_360_Y_40_: (**a**) Threefold, (**b**) Fourfold, (**c**) Fivefold, (**d**) Sixfold.

The percentages (PCTs) of the main local structural symmetries are measured to understand the structural ordering in liquid Mg-Y-X alloys. Figure 4 exhibits the PCT changes in the local structural symmetries from liquid Mg_360_Y_40_ to Mg_320_Y_40_X_40_. The counterparts in liquid pure Mg are also given. Compared with pure Mg, the liquid structure of Mg_360_Y_40_ alloys is composed of less threefold and fourfold symmetric local structures and more fivefold and sixfold ones, in other words, Y leads to the transformation of local structures from low symmetries to high. Accompanied by the addition of X (X = Co, Ni, Cu, Zn), the high symmetries are weakened, while the low ones are enhanced, reflecting that X-induced change in structural ordering is opposite to the Y-induced for Type I LPSO Mg-Y-X alloys.

**Figure 5:**
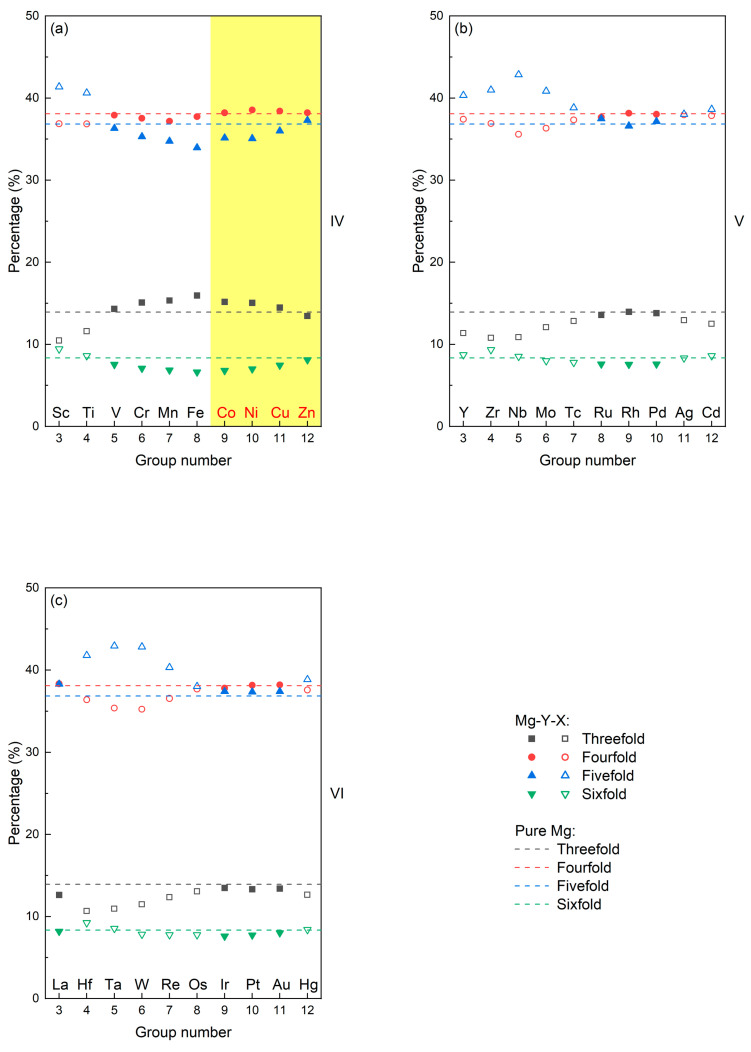
The distributions of main local structural symmetries in liquid Mg_320_Y_40_X_40_ alloys, which are compared with that in liquid pure Mg: (**a**) X in Period IV, (**b**) X in Period V, (**c**) X in Period VI.

Then, the distribution of the main local structural symmetries in liquid Mg_320_Y_40_X_40_ alloys is shown in Figure 5, which is compared with the circumstance in pure Mg. For liquid pure Mg, the fourfold and fivefold symmetries dominate the structural ordering, and the local structural symmetries are sorted in descending order by PCT as follows: PCT_fourfold_ > PCT_fivefold_ > PCT_threefold_ > PCT_sixfold_. It is worth noting that the distribution of the main local structural symmetries in pure Mg recurs in the reported Type I LPSO Mg-Y-X alloys (X = Co, Ni, Cu, Zn), which is contributed by the fact that X-induced change in structural ordering is opposite to the Y-induced.

Based on the results from equilibrium volume and cohesive energy, CSRO, and local structural symmetry, the common liquid characteristics of Type I LPSO Mg-Y-X alloys can be described as follows: X should result in the reduction in equilibrium volume and cohesive energy; Y should repulse Y and be attracted by both Mg and X, and X should be repulsed by both Mg and X; X should enhance the threefold and fourfold symmetries and weaken the fivefold and sixfold ones so that the local structural symmetries are distributed as PCT_fourfold_ > PCT_fivefold_ > PCT_threefold_ > PCT_sixfold_.

### 3.2. The Experimental Determination of New Type I LPSO Mg-Y-X Alloy

**Figure 6:**
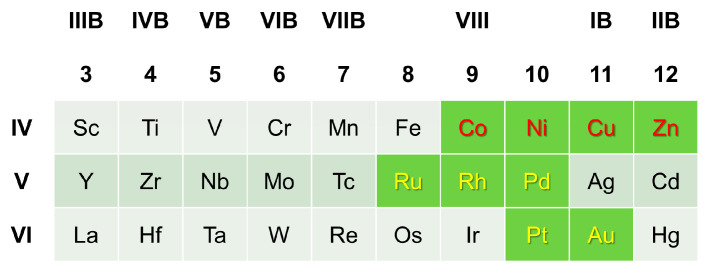
The X elements in the Mg-Y-X alloys satisfying all the common liquid characteristics of Type I LPSO Mg-Y-X alloys (X = Co, Ni, Cu, Zn), which are highlighted in yellow.

As exhibited in Figure 6, in terms of the fact that the Mg-Y-X alloy satisfies all the common liquid characteristics of Type I LPSO Mg-Y-X alloys (X = Co, Ni, Cu, Zn) and has not been reported with the LPSO phase, Mg-Y-X alloys (X = Ru, Rh, Pd, Pt, Au) can be identified as the candidate Type I LPSO alloy. To verify whether a new Type I LPSO Mg alloy can be confirmed from the candidate ones, the Mg-Y-Au alloy is selected for further experimental determination.

**Figure 7:**
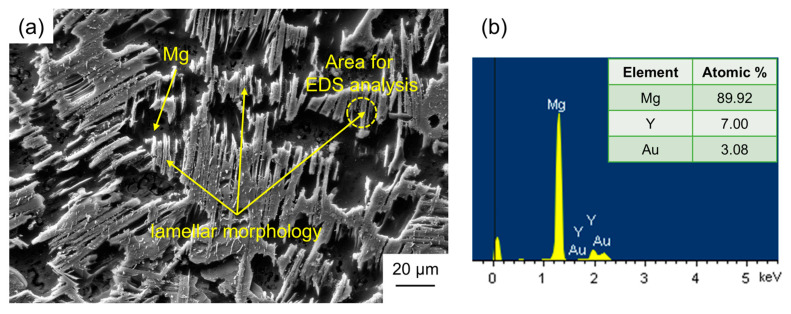
The SEM image (**a**) of the as-cast Mg_92.5_Y_5_Au_2.5_ alloy combined with the EDS analysis for the lamellar morphology (**b**).

The SEM image of the as-cast Mg_92.5_Y_5_Au_2.5_ alloy are shown in Figure 7, and α-Mg phase with black contrast is observed. More importantly, the lamellar morphology, consisting of 89.92 at. % Mg, 7.00 at. % Y, and 3.08 at. % Au according to the EDS analysis, is widely distributed on the grain boundaries or between the dendrite arms of α-Mg, which always indicates the formation of the LPSO phase [38,39,40].

**Figure 8:**
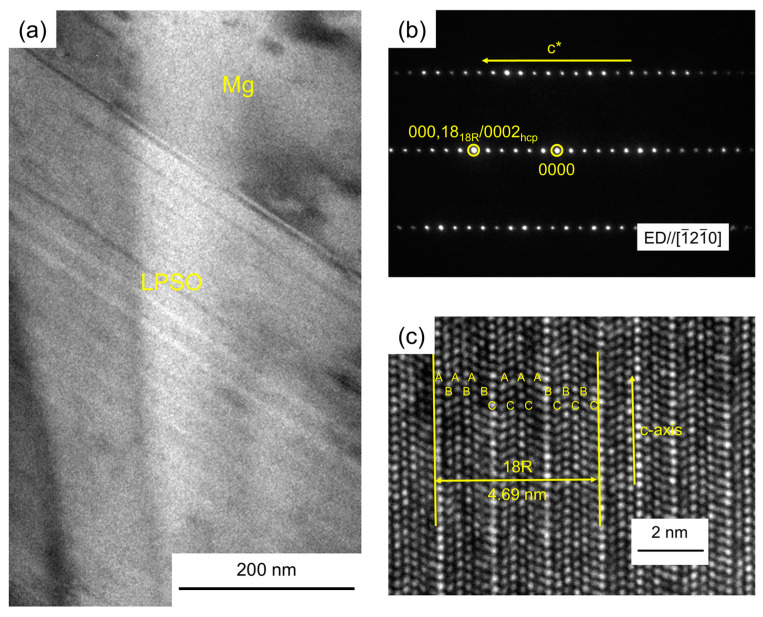
The bright-field TEM image (**a**) of the as-cast Mg_92.5_Y_5_Au_2.5_ alloy combined with the SAED pattern (**b**) and high-resolution TEM image (**c**) of the LPSO phase.

In order to determine the structure of lamellar morphology in the as-cast Mg_92.5_Y_5_Au_2.5_ alloy, the TEM images and corresponding SAED pattern are given in Figure 8. From the bright-field TEM image, a considerable number of platelets are observed in the phase with lamellar morphology, which form parallel lines to each other. The SAED pattern further shows that five extra diffraction spots appear between the transmission spot and (0002)_Mg_ along the [$\bar{1}$2$\bar{1}$0] zone axis, and the stacking sequence of ABABABCACACABCBCBC is identified in the high-resolution TEM image with the period of 4.69 nm. The above results demonstrate that the LPSO phase is formed in the as-cast Mg_92.5_Y_5_Au_2.5_ alloy with 18R structure [41], and therefore, the as-cast Mg-Y-Au alloy can be ultimately confirmed to be a Type I LPSO alloy.

### 3.3. The Liquid–Solid Correlation in Type I LPSO Mg-Y-X Alloy

**Figure 9:**
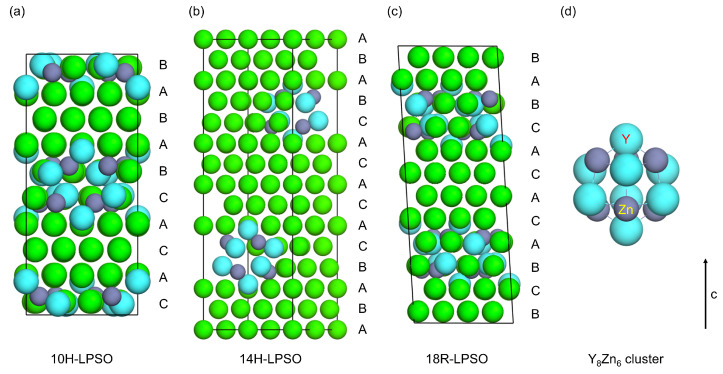
The crystal structures of the LPSO phases in Mg-Y-Zn alloy [42]: (**a**) 10H, (**b**) 14H, (**c**) 18R, (**d**) Y_8_Zn_6_ cluster.

The discovery of a Type I LPSO Mg-Y-Au alloy following the liquid characteristics returns a support for liquid–solid correlation. Taking the crystal structures of the LPSO phases in Mg-Y-Zn alloys as an example, the latest crystal structures to the best of our knowledge [42] are exhibited in Figure 9, including 10H, 14H, and 18R structures. The solute atoms are segregated as a Y_8_X_6_ cluster within four layers stacked along the c-axis, and Mg-Y, Mg-X, and Y-Zn pairs constituted by the nearest neighbors are enriched in the solute segregation layers. For the common liquid characteristics of Type I LPSO Mg-Y-X alloys, the Warren-Cowley parameters *α_Mg_*_-*Y*_ < 0, *α_Mg_*_-*X*_ > 0, *α_X_*_-*Y*_ < 0, *α_Y_*_-*Y*_ > 0, and *α_X_*_-*X*_ > 0 are obtained. *α_X_*_-*Y*_ is the most negative among all the parameters. The strong attraction of X to Y favors the formation of a Y-X pair in the liquid, in other words, solute segregation is able to occur even in the liquid. *α_Mg_*_-*Y*_ < 0 and *α_Mg_*_-*X*_ > 0 mean that Mg atoms prefer to attract Y rather than Zn, which is consistent with the pattern that the top and bottom of the solute segregation layers in the crystal structures of the LPSO phases are only constituted by Mg and Y, and there are more Mg-Y pairs than Mg-Zn ones. For *α_Y_*_-*Y*_ > 0 and *α_X_*_-*X*_ > 0, the repulsion between Y atoms and the repulsion between X atoms are manifested as the fact that Y and X cannot be their respective nearest neighbor in the Y_8_X_6_ cluster.

Further, X results in the reduction in equilibrium volume and simultaneously enhances the threefold and fourfold symmetries and weakens the fivefold and sixfold ones to make the local structural symmetries be distributed as PCT_fourfold_ > PCT_fivefold_ > PCT_threefold_ > PCT_sixfold_. The common liquid characteristics of Type I LPSO Mg-Y-X alloys demonstrate that X serves for offsetting the structural effect given by Y and vice versa, which leads the liquid structure of the Mg-Y-X alloy to approach pure Mg. From this point of view, the Y_8_X_6_ clusters in the crystal structures of the LPSO phases should be the atom arrangement mode alleviating the structural changes caused by the addition of solutes in HCP-Mg. The atomic size of Y is larger than Mg, while the atomic size of X is smaller, and the formation of Y_8_X_6_ clusters alleviates the volume change in the lattice by binding Y and X together. On the other hand, all the atom layers in the crystal structures of the LPSO phases are close-packed; what is more, most of the layers are stacked with the sequence of ABAB, and only a small amount of stacking faults are generated, owing to the formation of Y_8_X_6_ clusters. The structural ordering in HCP-Mg is preserved in the LPSO phases as much as possible, which reflects the alleviation of the structural ordering change.

Comprehensively, the common liquid characteristics obtained from the reported Type I LPSO Mg-Y-X alloys (X = Co, Ni, Cu, Zn) are capable of serving as the formation criteria of the LPSO phase from the perspective of liquid–solid correlation. In the description on the liquid characteristics, the employed parameters are preciously defined by AIMD simulation independent of ICSD. On this basis, the common liquid characteristics can be supplemented from all the reported Type I LPSO Mg alloys and continuously self-updated with the discovery of new ones to develop the formation criteria of the LPSO phase. That is the most important advantage in exploring the formation criteria from the perspective of liquid–solid correlation. Therefore, the liquid characteristics of Mg alloys are worthy of more extensive investigations to develop the formation criteria of the LPSO phase, which is valuable for accelerating the development of high-performance Mg alloys.

## 4. Conclusions

Aimed at exploring the formation criteria of the LPSO phase from the perspective of liquid–solid correlation, the common liquid characteristics of the reported Type I LPSO Mg-Y-X alloys (X = Co, Ni, Cu, Zn) are obtained by AIMD simulations in this work, and the discovery of a new Type I LPSO alloy is achieved according to the obtained liquid characteristics. The main conclusions are provided as follows:

(1) The common liquid characteristics of Type I LPSO Mg-Y-X alloys are described in three aspects: X should result in the reduction in equilibrium volume and cohesive energy; Y should repulse Y and be attracted by both Mg and X, and X should be repulsed by both Mg and X; X should enhance the threefold and fourfold symmetries and weaken the fivefold and sixfold ones so that the local structural symmetries are distributed as PCT_fourfold_ > PCT_fivefold_ > PCT_threefold_ > PCT_sixfold_.
(2) Besides the reported Type I LPSO Mg-Y-X alloys (X = Co, Ni, Cu, Zn), the Mg-Y-X alloys (X = Ru, Rh, Pd, Pt, Au) also satisfy all the common liquid characteristics and are predicted as the candidate Type I LPSO alloys. As the representative of the candidates, Type I LPSO Mg-Y-Au alloy is experimentally confirmed, in which the LPSO phase has 18R structure with the period of 4.69 nm.
(3) The discovery of a new Type I LPSO alloy supports the liquid–solid correlation. In the crystal structures of the LPSO phases, the atom distribution within the solute segregation layers is consistent with the CSRO in the liquid alloy, and Y_8_X_6_ clusters should be the atom arrangement mode alleviating the structural changes caused by the addition of solutes in HCP-Mg.

On this basis, it is worth pointing out that discovering more Type I LPSO Mg alloys according to the obtained common liquid characteristics and updating the common liquid characteristics relying on the discovered new Type I LPSO Mg alloys should be the guidelines for further research on the formation criteria of the LPSO phase from the perspective of liquid–solid correlation, which are valuable for accelerating the development of high-performance Mg alloys.

## Data Availability

The original contributions presented in the study are included in the article, further inquiries can be directed to the corresponding authors.
